# Formulation and Physicochemical Characterization of Cyclosporine Microfiber by Electrospinning

**DOI:** 10.15171/apb.2019.028

**Published:** 2019-06-01

**Authors:** Shahla Mirzaeei, Ghobad Mohammadi, Navid Fattahi, Pardis Mohammadi, Ali Fattahi, Mohammad Reza Nikbakht, Khosro Adibkia

**Affiliations:** ^1^Pharmaceutical Sciences Research Center, School of Pharmacy, Kermanshah University of Medical Sciences, Kermanshah, Iran.; ^2^Student Research Committee, Kermanshah University of Medical Sciences, Kermanshah, Iran.; ^3^Faculty of Pharmacy, Tabriz University of Medical Sciences, Tabriz, Iran.

**Keywords:** Bioavailability, Cyclosporine A, Electrospinning, Fibers, Polyethylene glycol, Povidone

## Abstract

***Purpose:*** The objective of this study was to improve the permeability and water solubility rate of a poor water soluble drug, cyclosporine A (CsA).

***Methods:*** In order to improve the drug dissolution rate and oral bioavailability, electrospinning method was used as an approach to prepare. The fibers were evaluated for surface morphology, thermal characterizations, drug crystallinity, *in vitro* drug release and *in vivo* bioavailability studies.

***Results:*** Scanning electron microscope (SEM) results confirmed that the fibers were in microsize range and the size of the fibers was in the rang of 0.2 to 2 micron. Differential scanning calorimetry (DSC) and powder X-ray diffractometry (XRPD) analysis ensured that the crystalline lattice of drug were weakened or destroyed in the fibers. The drug release was 15.28%, 20.67%, and 32.84% from pure drug, fibers of formulation B, and formulation A, respectively. *In vivo* study results indicated that the bioavailability parameters of the optimized fiber formulation were improved and the maximum concentration (C_max_) were significantly higher for fibers (3001 ng/mL) than for pure drug (2550 ng/mL). The dissolution rate of the formulations was dependent on the nature and ratio of drug to carriers.

***Conclusion:*** The physicochemical properties showed that the optimized mixture of polyethylene glycol (PEG) and povidone (PVP) fibers could be an effective carrier for CsA delivery. PEG and PVP fibers improved the absolute bioavailability and drug dissolution rate with appropriate physicochemical properties.

## Introduction

Cyclosporine A (CsA) is a neutral, lipophilic, cyclic endecapeptide that has been used clinically to treat systemic, local autoimmune disorders and prevent allograft rejection in various organ transplantations.^1,2^ CsA shows low dissolution rate in an aqueous medium that leads to the weak absolute bioavailability due to its low permeability and low watersolubility.^3,4^ Many different approaches have been used to improve the bioavailability and enhance the permeability of peptide drugs such as CsA.^4-6^

Drug delivery with micro/nanocarriers has gained huge interest because micro/nanocarriers can overcome many major problems that caused by conventional free drugs.^7^ Different methods have been used to fabricate micro/nanostructures for drug delivery purposes. Electrospinning is one of the best techniques for producing fibers in micro/nano rang with controlled surface morphology.^7,8^

The usage of electrospun fibers as suitable carriers for drug delivery systems has gained widespread interest in pharmaceutics.^9^ The micro/nanofibers are generated by application of a strong electric field on polymer solution held in a syringe with a capillary outlet.^10^ Drug loaded electrospun fibers offer many advantages such as controlled drug release, high encapsulation efficiency, high drug loading capacity, ease of production and cost effectiveness for pharmaceutical, simultaneous delivery of multiple drugs, and biomedical applications.^11^

A wide range of biodegradable biopolymers such as polyethylene glycol (PEG), povidone (PVP), can be electrospun as the base materials, including suture fibers and scaffolds, for the tissue engineering with specific fiber arrangement.^12^ They are biocompatible and biodegradable polymers that they can provide high efficiency in drug loading.^12^

The aim of the present research was increased permeability and water solubility of CsA. In this work, we designed microfiber carriers in which the immunosuppressive drug, CsA, was dispersed into polymers matrix. Different amount of CsA was dissolved in PEG and PVP solution and microfibers were fabricated from this mixture by electro spinning method. CsA has been incorporated into different ratios of PEG and PVP. The microfiber scaffolds were evaluated for the physicochemical properties, in vitro release study and vivo release study. The results were compared and the best quality of microfiber was selected as the optimum amount to achieve effective treatment.

## Materials and Methods

### Materials

CsA powder was purchased from Abcam (Cambridge, MA, USA). PEG with an average molecular weight of 20 000, PVP, polyvinyl alcohol (PVA) with an average molecular weight of 12 000, acetonitrile, sodium hydrogen phosphate, phosphoric acid, and sodium hydroxide were purchased from Merck (Darmstadt, Germany).

### Solution preparation and electrospinning experiments

The preparation of solution procedure of this study consisted of bellow steps:

PVP solution was prepared by dissolving PVP in ethanol overnight under constant stirring, and also PEG solution was prepared by fully dissolving with magnetic stirring PEG in chloroform solvent.

Formulation A was prepared by adding PVP solution (0.5 g PVP and 3 g ethanol), PEG solution (3.5 g PEG and 5 g chloroform), and 167.5 mg (25 mg/L) CsA powder and then the mixture was stirred at 25°C for 20 minutes. The same procedure was followed in preparing formulation B. Except for formulation B the amount of drug was 335 mg (50 mg/L). Formulation C was prepared by adding 167.5 mg CsA powder to mixing of PVP solution (1.5 g PVP and 3 ml ethanol) and PEG solution (2.5 g PEG and 5 g chloroform). For formulation D, the method used was the same as formulation C, except that the amount of drug was 335 mg (50 mg/L).

The morphology and performance of the electrospun fiber were affected by the electrospinning parameters.^2^ The process of electrospinning was performed by Electroris (Fanavaran Nanomeghyas, Tehran, Iran). The process parameters were optimized to achieve uniform micro/nanofibers. During the electrospinning process, a high voltage was used to create smooth fibers free of beads (25 kV) at a distance of 14.5 cm. If solvents were not completely eliminated from the formulations, films without a recognizable fibrous structure were obtained. Therefore, the tests were carried out at 45°C and the flow rate for each solution was 0.5 ml/h. The drum rotated at a speed of about 200 rpm. Before electrospinning, the polymer solutions were fed into a syringe, which was controlled by a syringe pump (Fanavaran Nanomeghyas, Tehran, Iran).

### SEM analysis

LEO 440i (Leo Electron Microscopy Ltd., Cambridge, UK) scanning electron microscope (SEM) was used to observe the morphology of fibers. The surface of samples was sputter coated with gold and observed on the SEM.

### DSC analysis

Calorimetric measurements were carried out by using a DSC (DSC-60, Shimadzu, Japan). Approximately 3 mg of samples were placed on the DSC pans (3 mg) made of aluminum pans and sealed. DSC traces were recorded between 20 and 260°C at a heating rate of 40°C/min.

### PXRD analysis

The X-ray diffraction patterns were recorded with an automated diffrac­tion system (Siemens, Model D5000, and Germany), using the nickel filtered Cu K*α* radiation (*λ =* 1.54050 A°). Diffractograms were run at scanning rate of 0.06° cm^-1^, over the range of 5-60° and with sampling intervals of 0.02.

### In vitro drug release and drug release kinetics

The in vitro drug release of the microfibers was determined by dialysis method. The concentration of released was determined by HPLC (Knauer, Mainz, Germany) method.^13^

One milligram CsA equivalent of various formulations were resuspended in 1 mL of phosphate buffer solution (0.l M) at pH 7.4 and placed in a dialysis bag (molecular weight cut off 12000 Da). Phosphate buffer solution (Dissolve 13.6 g of KH2PO4 and 0.4 g of NaOH in distilled water and dilute to 1 L, pH 7.4) was prepared according to the US Pharmacopeia. Ethanol was used as a co-solvent for the preparation of soluble cyclosporine.^14,15^ Each dialysis bag was placed in a flask containing 10 ml phosphate buffer and was incubated at 37°C in an incubator shaker (Titramax 1000, Heidolph, Germany) with speed of 100 rpm. At predetermined times (1, 3, 6, 12, 24, 48, 72, and 96 hours), 1 ml of samples were taken and replaced with fresh buffer while maintaining strict sink condition throughout the experiment.

HPLC separations were performed on a 150 × 4.6 mm C18 (pore size 5 μm) (Analyzentechnik, Mainz, Germany) analytical column. The mobile phase consisted of 0.25 mmol phosphate buffer at pH 4.5 and acetonitrile (80:20 v/v). The flow rate was maintained at 1.5 mL/min and the detection was performed at 210 nm. The concentration of drug in the samples reconstituted was calculated based on a linear regression calibration curve.

The kinetics of drug release was fitted into five types of mathematical models including Zero order, First order, Hixson-Crowell, Higuchi square root, and Peppas to find out the mechanism of drug release. The kinetic model which displayed maximum squared correlation coefficient (RSQ) and minimum prediction error (PE) was selected as the best kinetic model.

### Animal bioavailability study

The male rats (Pastor Institute, Iran) at 8-11 weeks of age (average weight 160 g) were used for animal experiments. Animals were randomly divided into two groups of six rats each. In the first group, each rat received 15 mg drug per kilogram of body weight in aqueous suspension and Second group received optimum formulation of drug in a volume of 15 mg drug per kilogram of body weight via gavage.

The blood samples (500 μL) were withdrawn at specific intervals (1, 2, 3, 4, 5, 6, 12, and 24) from the cerebral blood flow of each rat. The samples were placed in plastic EDTA (Ethylene diamine tetra acetic acid) tubes to stop coagulation by complication with EDTA.

### In vivo drug analysis

Radioimmunoassay technique was used to determine the CsA concentration with SP-Whole Blood CsA direct RIA kit by the commercial laboratory (Beckman Coulter Inc., Brea, CA).^16^ According to the commercial laboratory, an antibody to an analyte of interest was coated onto a microtiter plate. One hundred microliters of whole blood samples were added to each plate and remained at room temperature for 30-40 minutes after washing; the second antibody (Anti-CYCLO-Trac SP ImmunoSep) was added to all pellets and remained for 30-40 at room temperature. After final washing; the remaining pellet was counted for radioactive ^125^I in a gamma counter (Stratec, Birkenfeld, Germany).

## Results and Discussion

### SEM results

**Figure 1:**
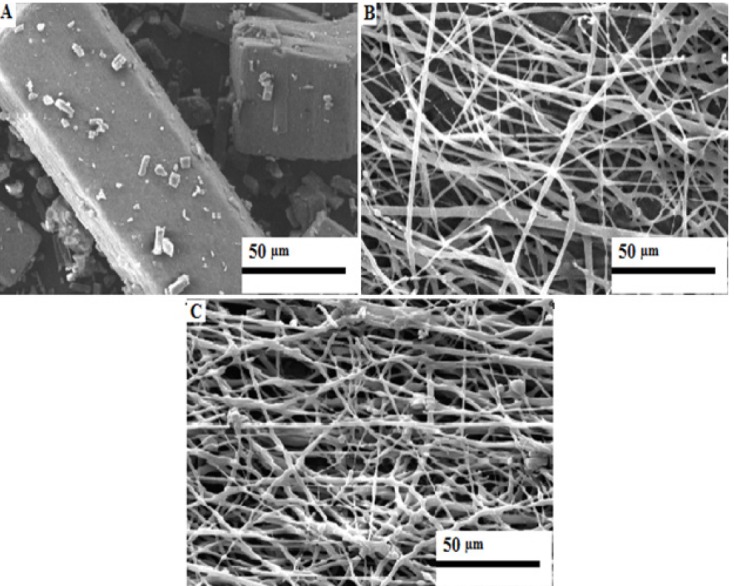
Scanning electron microscope (SEM) images of cyclosporine A (CsA) (A) prepared fiber via solution A (B) and B (C).

The SEM images of the fibers that were prepared via solution A and B are shown at Figure 1B and Figure 1C, respectively. The SEM results showed that microfibers were achieved. From the figures it is also clear that the size of the fibers was in the range of 0.2 to 2 micron with high surface area/volume ratios. These two properties could be lead to the higher drug loading and better drug dissolution rate. The same results were seen in the other researchs.^17^

The fibers were not successfully fuse and aggregate by electrospinning solution C and D during different electrospinning conditions. This is due to incomplete solvent evaporation as well as the collected fibers stuck to the collector.^18^

### DSC results

**Figure 2:**
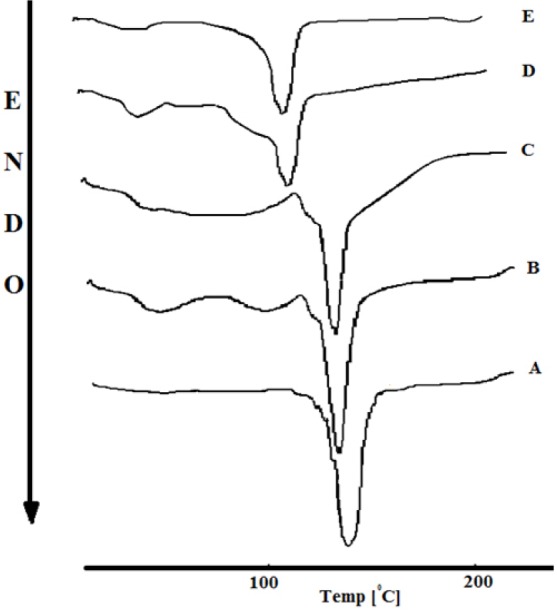
Differential Scanning Calorimetry(DSC)curve of the intact Cyclosporine A (CsA) powder (A) , physical mixtures of solution A (B) physical mixtures of solution B (C), fiber of solution A (D), and fiber of solution B (E).

DSC thermograms of the intact CsA, PEG, PVP, physical mixture and fibers are shown in Figure 2. Endothermic peaks at 139.51°C, 136.07­°C, and 111.28­°C is related to the melting point of CsA in the drug powder, physical mixture, and the fibers thermograms of formulation A (25 mg/ ml drug to polymer), respectively. Similar results were obtained for the fibers that prepared with formulation B. Endothermic peaks at 138.1°C and 120.66°C is related to the melting point of CsA in physical mixture, and the fibers thermograms of formulation B. Melting point reduction is a colligative property and therefore, Physical mixing of the drug with polymers resulted in the drug melting point reduction in the physical mixtures.^19^ The fibers exhibit the lowest melting point for CsA that may be due to the reduction in the crystalline behavior of drug in the fibers.^20,21^ Similar results were obtained for solid dispersions of piroxicam with hydrophilic carriers by Valizadeh et al^22^ DSC thermograms show that increasing of the drug to polymer ratio, in the physical mixtures and fibers, resulted in the increases of the drug melting temperature. These results could be due to the increasing of the samples crystallinity. The influence of drug/polymer ratio on the results of DSC analysis was in agreement with the results obtained by the DSC results of Verreck et al on the fibers containing amorphous drug dispersions generated by electrostatic spinning.^23^

According to the DSC results, the melting point of CsA in physical mixture and fibers of formulation B is higher than the formulation A. This can be attributed to the reductions of polymer amounts in formulation B. Colligative properties and the effect of polymer as a carrier to reduce the crystallinity were decreased with decreasing polymer amounts. Similar result has been obtained for the CsA particles by formulating the drug with mannitol as a hydrophilic matrix former.^15^

### XRPD results

**Figure 3:**
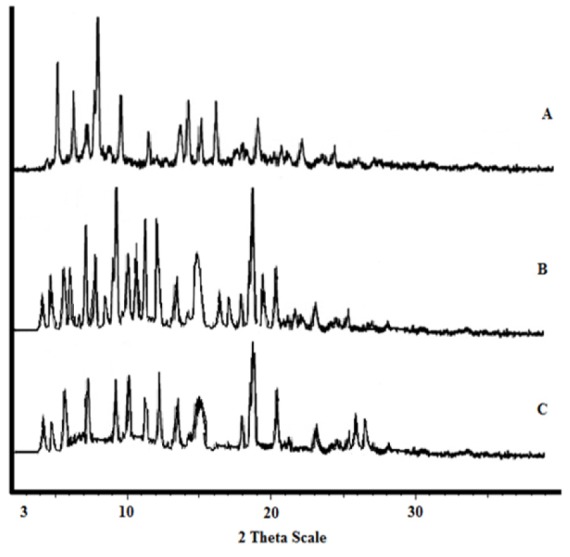
X-ray powder diffractions of cyclosporine A (CsA) powder (A) , physical mixtures of solution A (B) and fiber of solution B (E).

X-ray diffraction photographs of CsA powder, physical mixture and the fibers of optimum formulations are shown in Figure 3. According to the SEM photographs and DSC results, the formulation A is the optimum formulation. The XRD patterns of intact CsA powder revealed several distinct diffraction peaks in the 2°θ at 5, 6, 8, 10, 13, 14, and 15 that are indicative of CsA crystalline form.^24-26^ The crystalline form of CsA observed in the XRD analysis confirmed the results of DSC. Because of the endothermic peak at 139.51°C in DSC thermograms relating to crystalline form of CsA. The XRD photographs of physical mixture show sharp peaks at 5, 7, 9, 19, and 25 in the 2^o^θ which related to PEG and PVP polymers. The X-ray peaks of the optimum formulation were similar to the physical mixture but all peaks were smaller than the peaks of physical mixture. This result confirmed that degree of crystallinity of CsA was reduced. This could be the reason for the observed increase in the dissolution rate of the drug in the fibers.^27^

### In vitro release study

**Figure 4:**
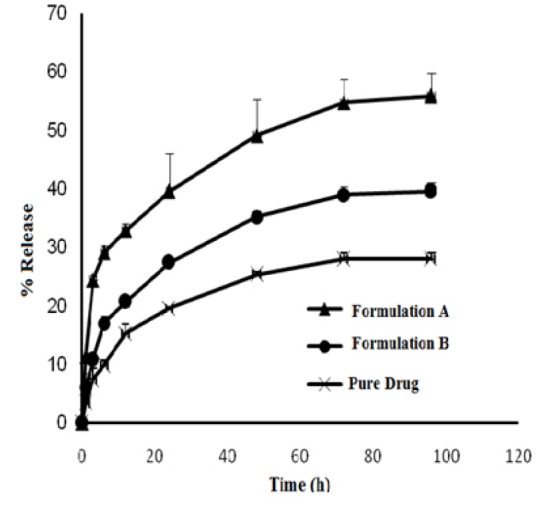
Dissolution profiles of Cyclosporine A powder, fiber of formulation A and B.

The *in vitro* drug release profiles for intact CsA powder and the formulated fibers are shown in Figure 4. The release percent of CsA from the fibers has been found to be faster in comparison with pure drug. In the first 12 hours, the percent of the drug release was 15.28%, 20.67%, and 32.84% from pure drug, fibers of formulation B, and formulation A, respectively. The relative increase in release rate observed for fibers by the calculation of the slope of in vitro release rate curves. This increase of dissolution rate could be the reason for the increase of the bioavailability parameters of drug (Table 1). Fibers of formulation B, revealed slower drug release rate in comparison with the fibers of formulation A. Increasing the amount of drug, at constant amount of polymers, resulted in a decrease of the CsA release rate. This result is in agreement with the results reported by Donnell and McGinity.^28^ In vitro release results indicated that the decrease of crystalinity of drug in fibers, increase of the drug hydrophilicity (due to increasing amount of PVP and PEG hydrophilic polymers), and high surface area/volume ratios of fibers could be the reasons for increasing the release rate of fibers.

**Table 1:** Pharmacokinetic parameters for pure drug powder and optimized formulation with relative Mean Standard Deviations (± SD)

**Formulation**	**Pure cyclosporine A**	**Optimized formulation**
AUC (ng.h/mL)	9587.91‏±181	10602 ± 98
C_max_ (ng/mL)	2550‏± 187	3001‏± 85
t_max_ (h)	3.5	3

To clarify the mechanism of release, the release data of microfiber formulations were fitted into various kinetic models. The prediction ability of the kinetic models was compared by calculation of squared correlation coefficients (RSQ) and percent error (PE). Considering the RSQ and PE values, Higuchi model resulted the best fitting between model for pure drug and formulation B and Peppas was the best kinetic model for formulation A.

### In vivo study

Optimized formulation and the pure CsA powder were chosen for in vivo evaluation. The whole blood CsA concentrations of optimized formulation and the pure CsA powder for each rat at interval times were calculated. The results of the relevant pharmacokinetic parameters for pure drug powder and optimized formulation including, the area under the plasma concentration-time curve (AUC), maximum concentration (C_­max_) and time to maximum concentration (t_max_) are listed in Table 1. Bioavailability of optimized formulation in rats was compared with pure drug by comparison of AUC values. Extent and rate of the bioavailability of optimized formulation was significantly greater than the mean bioavailability parameters of the pure drug. The enhanced bioavailability of CsA was due to the increased absorption by increasing the drug release rate. Results showed that the C_max_ were significantly higher for fibers (3001 ng/mL) than the pure drug (2550 ng/mL). Additionally, t_max_value of the pure drug (3.5 hours) was improved in the electro spun fibers (3 hours). The rate of absorption was obtained by calculating of the initial slope of the blood concentration versus time curve. The initial rate of absorption in the first 3 h was obtained directly from the plasma concentration curve. The higher rate of absorption in the fibers can lead to an increase of bioavailability. The average standard deviation of drug absorption for the optimized formulation was about 1.5 times lower than the case of the pure drug powder. These results show that the variations in the rate of bioavailability was higher for the fibers than for the pure drug. Similar results were obtained with CsA particles in comparative work reported by Shabouri et al^29^ and also, the results are in close agreement with the results for organic soils obtained by Bremner and Shaw.^7^

## Conclusion

The electrospinning process was used successfully to fabricate CsA-polymer microfibers. Several formulations were investigated to find the optimized one for the delivery of CsA. The high dissolution rate of CsA was obtained from fibers composed of 167.5 mg drug in the PVP to PEG with the ratio 1:7 (formulation A). The optimized CsA fibers shows significantly higher AUC, higher C_max_ and better t_max_ than the pure CsA powder. It could be due to the increase of CsA dissolution rate in the fibers. The results show that the bioavailability of drugs loaded in fibers was dependent on the physicochemical properties of drug. The selected optimized formulation, showing improved bioavailability of CsA, would be useful to deliver a poorly water-soluble CsA and could be applicable to the other poorly water-soluble drugs.

## Ethical Issues

All the experimental works were approved by the Ethical Committee of Kermanshah University of Medical Science (Kermanshah, Iran), number (KUMS .REC.1395.293) and conform to the European Communities Council Directive of 24 November 1986.

## Conflict of Interest

Conflicts of interest: none

## Acknowledgments

The authors wish to thank Kermanshah University of Medical Sciences, Kermanshah, Iran for the financial support of this research.
